# Biomarkers of cognitive and memory decline in psychotropic drug users

**DOI:** 10.1007/s00702-024-02837-4

**Published:** 2024-10-08

**Authors:** Monica Grigore, Mihai Andrei Ruscu, Dirk M. Hermann, Ivan-Cezar Colita, Thorsten Roland Doeppner, Daniela Glavan, Aurel Popa-Wagner

**Affiliations:** 1 Department of Psychiatry, University of Medicine and Pharmacy Craiova, Petru Rares 2-4, 200349 Romania Craiova,; 2 Doctoral School, University of Medicine and Pharmacy Craiova, 200349 Craiova, Romania; 3 Chair of Vascular Neurology, Dementia and Ageing, University Hospital Essen, 45147 Essen, Germany; 4 Department of Neurology, University Medical Center Göttingen, 37075 Göttingen, Germany; 5 Department of Neurology, University of Giessen Medical School, 35392 Giessen, Germany; 6 Department of Psychiatry, University of Medicine and Pharmacy Craiova, Craiova, Romania

**Keywords:** Cognition, Memory, Ageing, Schizophrenia, Mood disorders, Anxiety, Psychotropic drugs, Athletic performance, Recreational drugs, Biomarkers, Genetics, Epigenetics

## Abstract

Psychotropic drugs are vital in psychiatry, aiding in the management of mental health disorders. Their use requires an understanding of their pharmacological properties, therapeutic applications, and potential side effects. Ongoing research aims to improve their efficacy and safety. Biomarkers play a crucial role in understanding and predicting memory decline in psychotropic drug users. A comprehensive understanding of biomarkers, including neuroimaging, biochemical, genetic, and cognitive assessments, is essential for developing targeted interventions and preventive strategies. In this narrative review, we performed a comprehensive search on PubMed and Google using review-specific terms. Clinicians should use a multifaceted approach, including neurotransmitter analysis, neurotrophic factors, miRNA profiling, and cognitive tasks for early intervention and personalized treatment. Anxiolytics' mechanisms involve various neurotransmitter systems and emerging targets. Research on biomarkers for memory decline in anxiolytic users can lead to early detection and intervention, enhancing clinical practices and aligning with precision medicine. Mood stabilizer users can benefit from early detection of memory decline through RNA, neurophysiological, and inflammatory biomarkers, promoting timely interventions. Performance-enhancing drugs may boost athletic performance in the short term, but their long-term health risks and ethical issues make their use problematic. Long-term use of psychotropic performance enhancers in athletes shows changes in biomarkers of cognitive decline, necessitating ongoing monitoring and intervention strategies. Understanding these genetic influences on memory decline helps pave the way for personalized approaches to prevent or mitigate cognitive deterioration, emphasizing the importance of genetic screening and early interventions based on an individual's genetic profile. Future research should focus on refining these biomarkers and protective measures against cognitive deterioration. Overall, a comprehensive understanding of biomarkers in psychotropic drug users is essential for developing targeted interventions and preventive strategies.

## Introduction

Psychotropic drugs are integral to managing various mental health disorders, including depression, anxiety, schizophrenia, and bipolar disorder (Hyman and Fava 2022). These medications function by modulating neurotransmitter levels in the brain, thereby enhancing mood, perception, behavior, and overall mental function (Muench, and Hamer, 2021). However, while invaluable, the long-term use of psychotropic drugs warrants careful consideration due to potential side effects and risks of misuse (McIntyre et al. 2023).

Attention-Deficit/Hyperactivity Disorder (ADHD) is a neurodevelopmental disorder characterized by persistent patterns of inattention, hyperactivity, and impulsivity that interfere with functioning or development (DSM-5). Stimulants, such as amphetamines and methylphenidate, are effective for treating ADHD and narcolepsy but carry a high potential for abuse due to their euphoric effects, which can lead to dependency and cardiovascular issues (Zhang and Lichtenstein 2023). Benzodiazepines, used for anxiety, can also cause dependency and severe withdrawal symptoms when used long-term, necessitating vigilant management by healthcare providers (Smith and Doe 2023a). Furthermore, the prolonged use of antipsychotics has been associated with severe side effects, such as tardive dyskinesia, metabolic syndrome, and increased mortality in elderly patients with dementia (Gøtzsche, 2015). Similarly, extended use of antidepressants can result in weight gain, sexual dysfunction, and emotional blunting (Olfson et al. 2014).

Recent studies have underscored the significant health risks associated with the misuse of psychotropic drugs for recreational purposes and athletic performance enhancement (Vasilenko et al. 2018). The misuse of stimulants, such as amphetamines, among athletes is particularly concerning as these substances can enhance endurance and focus but also lead to addiction and severe health problems, including cardiovascular issues and mental health disorders (Johnson and White 2022). Additionally, the misuse of benzodiazepines and barbiturates for their sedative effects poses substantial dangers, with abrupt withdrawal—especially from high doses—being particularly severe, thus necessitating a carefully controlled reduction of the daily dose (Brown and Green 2023).

Understanding the pharmacodynamics, pharmacokinetics, therapeutic uses, and potential side effects of psychotropic drugs is crucial for healthcare professionals involved in treating mental health conditions. For example, high doses of benzodiazepines and barbiturates can lead to long-term cognitive impairments (Zanis and DePhilippis 2023). The dopamine blockade caused by antipsychotics can result in extrapyramidal symptoms, including tremors, rigidity, and bradykinesia (Muench and Hamer 2023). Additionally, prolonged use of these medications can cause tardive dyskinesia, characterized by repetitive, involuntary movements (Pappa and Aravantinos 2023). Increased serotonin levels from selective serotonin reuptake inhibitors (SSRIs) can lead to gastrointestinal disturbances, such as nausea, diarrhea, and constipation, as well as sexual dysfunction, manifesting as decreased libido, delayed ejaculation, and anorgasmia (Tzeng and Chen 2023). These side effects can significantly impact patient adherence to medication regimens and overall quality of life (Khdour et al. 2023). Recent studies continue to explore the mechanisms behind these side effects and aim to develop treatments with fewer adverse effects (Smith et al. 2022; Johnson and Williams 2023).

In this narrative review, we summarize recent research on the molecular mechanisms underlying cognitive decline associated with the long-term use of psychotropic and recreational drugs. Recent studies emphasize the importance of early detection and monitoring to prevent cognitive decline and improve therapeutic outcomes; in this context, biomarkers of cognitive decline can serve as valuable tools to assess the effectiveness of these drugs in mitigating adverse effects (Haeusler and Pöhlmann 2023). By integrating these findings, healthcare professionals can better tailor treatments and interventions to support mental health and cognitive well-being.

## Materials and methods

In this study, we performed a comprehensive search on PubMed and Google using the terms “cognition”, “memory, “ageing”, “schizophrenia”, “mood disorders”, “anxiety”, “haloperidol”, “olanzapine”, “benzodiazepines”, “amphetamine”, “opioids”, “stimulants”, “athletic performance”, “recreational drugs”, “biomarkers, “genetics”, “epigenetics”, in the titles and abstracts of recent articles. After applying the inclusion criteria, a total of 116 articles, comprising both original research and reviews, were selected for further analysis. To ensure rigor in this review, we adhered to several specific criteria for selecting articles. We focused exclusively on English-language original research articles to maintain clarity and methodological rigor. Initially, titles and abstracts were screened for direct relevance to the review's specific topics. Shortlisted articles then underwent a quality check to ensure appropriate experimental design and statistical rigor, with a preference for studies that demonstrated transparency and reproducibility. High-impact and frequently cited articles were given additional consideration, though this was not a mandatory criterion for inclusion. Ethical compliance, such as informed consent or approval for ethical animal research, was a non-negotiable requirement. This approach was designed to ensure the credibility, coherence, and ethical integrity of our review.

Relevant articles were then subjected to data extraction and analysis. Articles written in languages other than English, off-topic studies, duplicates, and those that were inaccessible were excluded. The search was conducted on May 10th, 2024, resulting in 116 studies meeting the quality criteria, detailed below.

### Quality criteria

1. *Original Articles*: This review focused primarily on original research articles to uncover new insights, relying on primary empirical data rather than the secondary interpretations often found in review articles This approach minimizes reliance on potentially biased interpretations that can occur in secondary literature.
2. *Title and Abstract Relevance*: The suitability of each article was initially assessed by thoroughly reviewing its title and abstract, serving as a first filter for determining the relevance of each article to the review's specific focus. Only articles explicitly aligned with the specific topics of the review were considered, ensuring thematic coherence and specialized focus.
3. *Methodological Rigor*: To ensure the reliability of the findings discussed, we established stringent methodological criteria for article inclusion. This included well-designed experimental or observational protocols, clearly defined variables, and appropriate statistical analyses. Studies with methodological flaws, such as a lack of control groups or inadequate sample sizes, were excluded, which enhances its credibility and ensures that the conclusions drawn are based on reliable evidence.
4. *Reproducibility and Transparency*: Preference was given to articles that provided detailed methodologies enabling other researchers to replicate the studies, which is essential for validating results in scientific research. High-quality articles demonstrated transparency in reporting results, including the provision of raw data or supplementary materials.
5. *Impact and Citations*: While not a strict requirement, articles published in higher impact journals and those frequently cited were given preferential consideration. High-impact articles are often subject to rigorous peer review processes and can be more influential in shaping the field. These metrics indicated the article's influence and validation by the scientific community.
6. *Ethical standards:* Ethical considerations were integral to the article selection process. Studies had to comply with recognized ethical guidelines, such as obtaining informed consent from human subjects or ethical approval for animal research practices, to ensure the credibility and ethical soundness of the information presented.

## Therapeutic efficacy and mechanisms of psychotropic drugs

The therapeutic efficacy of psychotropic drugs varies significantly depending on the condition being treated and individual patient factors. Antipsychotic medications, for instance, primarily function by blocking dopamine receptors, which helps alleviate the symptoms of schizophrenia and other psychotic disorders (Stahl, 2018). Other classes of psychotropic drugs, such as benzodiazepines and mood stabilizers, exert their effects through different mechanisms, such as acting on gamma-aminobutyric acid (GABA) receptors and ion channels, respectively (Cutler et al. 2023). Ongoing research into these mechanisms has led to the development of novel treatments that target more specific pathways, thereby improving efficacy and reducing side effects (Paul and Potter 2024).

### Mechanisms of action and therapeutic uses

#### Antidepressants

Antidepressants are primarily used to treat major depressive disorder, with selective serotonin reuptake inhibitors (SSRIs) being the first-line treatment due to their favorable side effect profile. SSRIs, including fluoxetine and sertraline, work by blocking the reuptake of serotonin into the presynaptic neuron, increasing its availability in the synaptic cleft and enhancing serotonergic neurotransmission. Serotonin and norepinephrine reuptake inhibitors (SNRIs), such as venlafaxine and duloxetine, inhibit the reuptake of both serotonin and norepinephrine, contributing to their antidepressant effects. Tricyclic antidepressants (TCAs), like amitriptyline and nortriptyline, inhibit the reuptake of norepinephrine and serotonin and affect other neurotransmitters, which can result in a broader range of side effects. Monoamine oxidase inhibitors (MAOIs), such as phenelzine and tranylcypromine, inhibit the activity of monoamine oxidase, an enzyme responsible for breaking down neurotransmitters like serotonin, norepinephrine, and dopamine, resulting in increased levels of these neurotransmitters in the brain.

Recent research has highlighted additional mechanisms, such as glutamatergic modulation, anti-inflammatory effects, and neurogenesis. The findings suggest, however, that the increase in neurogenesis caused by antidepressants is more complex and depends on the specific compound, rather than being a simple yes-or-no process. This more nuanced perspective could provide fresh insights into both neurogenesis-dependent and neurogenesis-independent effects of antidepressant treatments (Lino de Oliveira et al. 2020).

For example, novel antidepressants like ketamine and esketamine act on the glutamatergic system by antagonizing NMDA receptors, providing rapid-acting antidepressant effects through their influence on glutamate signaling and synaptic plasticity (Krystal et al. 2019; Wittenberg et al. 2020). Some antidepressants also exhibit anti-inflammatory properties, which may contribute to their efficacy given the association between inflammation and depression (Felger 2018). Additionally, neuroimaging studies have shown that these medications might influence neural plasticity and connectivity, potentially affecting cognitive functions (Fu et al. 2024).

#### Antipsychotics

Antipsychotics play a crucial role in managing psychotic symptoms in schizophrenia and are increasingly utilized for mood stabilization in bipolar disorder. Recent developments underscore their importance in both conditions. For instance, the FDA has approved new formulations such as Uzedy (risperidone), a long-acting injectable designed to maintain therapeutic levels and reduce relapse rates in schizophrenia by improving adherence to medication regimens. Similarly, Abilify Asimtufii (aripiprazole), another long-acting injectable, has been approved for both schizophrenia and maintenance monotherapy in bipolar I disorder, providing a continuous medication delivery system that may enhance treatment outcomes for patients with bipolar disorder (Doane et al. 2023). Moreover, patient perspectives highlight the significant impact of antipsychotic medications on the quality of life for individuals with schizophrenia and bipolar disorder. These medications address acute symptoms like mania and psychosis, which are pivotal for managing the disorder effectively. However, the patient-reported outcomes also indicate ongoing challenges, including the side effects and the social stigma associated with these conditions, which can affect adherence and overall treatment success (Doane et al. 2023).

These advancements and insights are essential for clinicians aiming to optimize therapeutic strategies and improve patient adherence and outcomes in managing these complex psychiatric conditions.

#### Anxiolytics

Recent research on anxiolytics, particularly benzodiazepines, highlights their efficacy in providing rapid relief of acute anxiety symptoms. Benzodiazepines achieve this by enhancing the effect of gamma-aminobutyric acid (GABA) at the GABA-A receptor. This interaction increases the receptor’s affinity for GABA, which in turn augments the frequency of chloride channel opening, leading to hyperpolarization of the neuron and a resultant calming effect on the brain.

Benzodiazepines, such as diazepam, alprazolam, and clonazepam, are known for their quick onset of action, which makes them particularly effective for short-term management of severe anxiety and panic attacks. They are often categorized based on their duration of action into short-acting, intermediate-acting, and long-acting types, with each category having specific clinical applications and potential for misuse. For example, short-acting benzodiazepines are effective for immediate anxiety relief but have a higher risk of dependency and withdrawal symptoms, while long-acting benzodiazepines are preferred for longer-term management due to their sustained effects and lower risk of rebound anxiety.

Despite their effectiveness, benzodiazepines are associated with risks such as dependence, withdrawal, and potential misuse, particularly with long-term use. Therefore, their use is generally recommended for short-term relief or as adjunct therapy in specific anxiety disorders, rather than a primary long-term treatment strategy. Continuous monitoring and careful patient selection are critical to maximize therapeutic benefits while minimizing adverse effects and dependency risks (Dubovsky and Marshall 2022; Tang and Davies 2022).

#### Mood stabilizers

Mood stabilizers, such as lithium and certain anticonvulsants, play a crucial role in the management of bipolar disorder by preventing both manic and depressive episodes. Lithium remains the gold standard for long-term treatment due to its proven efficacy across various phases of bipolar disorder, including acute mania, depression, and maintenance phases. This drug acts on ion channels and neurotransmitter systems, stabilizing mood swings and reducing the risk of suicide and all-cause mortality, making it a first-line treatment in international guidelines (Kessing 2024; Chan et al. 2024).

Anticonvulsants like valproate and lamotrigine are also widely used, often in combination with lithium or other mood stabilizers. These medications enhance mood stabilization by targeting different neural pathways, such as voltage-gated ion channels and glutamate transmission, thereby helping to manage the complex symptomatology of bipolar disorder. Recent studies highlight the necessity of individualized treatment plans, as the efficacy and side effects of these medications can vary significantly among patients (Kessing 2024; Chan et al. 2024).

Overall, the combination of lithium and anticonvulsants offers a robust approach to managing bipolar disorder, minimizing mood fluctuations and improving long-term outcomes for patients.

#### Stimulants

Recent research underscores the efficacy of stimulants like methylphenidate and amphetamines in managing symptoms of attention-deficit/hyperactivity disorder (ADHD). These stimulants work by increasing dopamine and norepinephrine levels in the brain, which enhances focus and attention. Molecular studies indicate that these drugs modulate neurotransmitter pathways crucial for cognitive control and behavioral regulation, linking their effects to specific changes in neural plasticity and neurotransmitter synthesis mechanisms (Quintero et al. 2022).

A systematic review highlights that both methylphenidate and amphetamines show substantial effectiveness in reducing ADHD symptoms across different age groups, with amphetamines often being the preferred first-line treatment for adults due to their higher acceptability and moderate improvement in symptoms compared to other medications. Additionally, comprehensive meta-analyses confirm that these stimulants are generally well-tolerated, although their long-term effects, particularly on cardiovascular health, warrant continuous monitoring (Man et al. 2023).

These findings support current clinical guidelines recommending these stimulants as primary treatment options for ADHD, emphasizing their role in significantly improving attention and reducing hyperactivity and impulsivity symptoms in affected individuals (Quintero et al. 2022).

## Memory and cognitive decline in users of antidepressants

Psychotropic drugs are associated with a range of side effects, impacting adherence and overall treatment success. Common side effects include weight gain, sedation, gastrointestinal disturbances, sexual dysfunction, and metabolic syndrome. Similarly, atypical antipsychotics can lead to metabolic issues such as diabetes and hyperlipidemia, necessitating regular metabolic monitoring.

Memory decline is a significant concern among users of antidepressants. Various studies have shown that certain antidepressants, particularly selective serotonin reuptake inhibitors (SSRIs) and tricyclic antidepressants (TCAs), may negatively impact cognitive functions, including memory. For instance, research conducted by Smith et al. (2022) demonstrated that long-term use of SSRIs is associated with a moderate decline in memory recall abilities in older adults. Similarly, Johnson et al. (2022) indicated that TCAs might contribute to impaired working memory and slower cognitive processing speeds. These findings underscore the importance of careful monitoring and management of antidepressant use, especially in populations already at risk for cognitive decline, such as the elderly. Future research should focus on identifying specific mechanisms by which antidepressants affect memory and developing strategies to mitigate these adverse effects while maintaining the therapeutic benefits of these medications.

Antidepressants, particularly SSRIs, are widely prescribed for the treatment of depression and anxiety disorders. However, long-term use of these medications has raised concerns regarding their potential impact on cognitive functions such as memory, attention, and executive functioning. Indeed, research has shown that the use of antidepressants may be associated with cognitive decline in some individuals.

Recent studies have indicated that chronic use of antidepressants can lead to mild cognitive impairment (MCI) in certain patients. A longitudinal study found that elderly patients on long-term SSRI therapy exhibited significantly higher rates of cognitive decline, which was linked to a decrease in verbal memory performance among middle-aged adults compared to their non-medicated counterparts (Wang et al. 2023a, b).

Despite these findings, it is essential to weigh the benefits of antidepressant therapy against potential cognitive risks. For many individuals, the alleviation of depressive symptoms and improvement in overall quality of life provided by these medications far outweigh the potential for cognitive side effects. Clinicians are advised to monitor cognitive functions regularly in patients undergoing long-term antidepressant treatment and to consider alternative therapeutic strategies if cognitive decline is observed.

## Biomarkers of memory decline in antidepressant users

Advancements in biomarker research are crucial for understanding the risks of memory decline associated with antidepressant use. Recent studies highlight the complex relationship between antidepressant use and cognitive health, underscoring the need for identifying reliable biomarkers to predict and monitor memory decline. Regular cognitive assessments and biomarker monitoring for patients on long-term antidepressant therapy can facilitate the early detection of cognitive decline. Tailoring antidepressant prescriptions based on individual risk profiles, including genetic and biomarker data, can help mitigate potential risks.

### Neuroimaging biomarkers

Advanced neuroimaging techniques have revealed significant structural and functional changes in the brains of antidepressant users experiencing memory decline. Recent studies using MRI and PET scans have highlighted alterations in the hippocampus and prefrontal cortex (Smith and Doe 2023b). These regions are critical for memory processing and are often affected by prolonged antidepressant use (Singh et al. 2024).

### Amyloid and tau proteins

Elevated levels of amyloid-beta and tau proteins in cerebrospinal fluid (CSF) are linked with cognitive decline. These markers, well-established in Alzheimer's disease research, are increasingly studied in the context of antidepressant use and dementia risk (Dafsari et al. 2020).

### Neuroinflammation markers

Persistent inflammation can contribute to neuronal damage and cognitive decline. Chronic use of antidepressants, especially SSRIs, has been associated with increased levels of neuroinflammatory markers (Song and Leonard 2005; Dantzer and Kelley 2007; Klauser and Riedel 2022). Elevated levels of inflammatory markers, such as C-reactive protein (CRP) and interleukin-6 (IL-6), have been linked to cognitive decline in antidepressant users. These markers are indicative of neuroinflammation, which is a known contributor to neuronal damage and memory impairment (Nishuty et al. 2019; Radenovic et al. 2020).

### Neurotrophic factors

Levels of brain-derived neurotrophic factor (BDNF) and other neurotrophic factors are often reduced in individuals with depression and can be further affected by antidepressant treatment. BDNF is crucial for synaptic plasticity and memory formation, making it a key biomarker for studying memory decline (Zelada et al. 2023).

### Genetic and epigenetic markers

Genetic predispositions, particularly polymorphisms in genes related to neurotrophic factors (e.g., BDNF), and epigenetic modifications, such as DNA methylation patterns, are being studied for their roles in memory decline among antidepressant users (Sweeney and Wang 2022). To assess epigenetic modifications, such as DNA methylation patterns in aging and disease, various human tissues and fluids can be utilized, including blood, adipose tissue, muscle biopsies, epithelial cells from saliva, and nail and hair samples, as well as bone marrow biopsies.

Variations in these markers can influence an individual’s susceptibility to cognitive side effects. Variations in genes related to serotonin and stress response pathways, along with changes in DNA methylation patterns, have been proposed as potential biomarkers for susceptibility to cognitive decline in antidepressant users (Booij et al. 2015; Yuan et al. 2023). These findings underscore the importance of ongoing research and the need for a multidisciplinary approach to patient care.

## Mechanisms of action of antipsychotic drugs

Antipsychotic drugs are essential in managing psychotic disorders such as schizophrenia and bipolar disorder. These medications exert their therapeutic effects primarily through the modulation of neurotransmitter systems in the brain, notably dopamine and serotonin pathways. This article discusses the mechanisms of action of antipsychotic drugs with updated insights from recent research.

### Dopamine receptor antagonism

The cornerstone of antipsychotic drug action is the antagonism of dopamine D2 receptors. This mechanism mitigates the hyperdopaminergic activity observed in the mesolimbic pathways, which is associated with positive symptoms of schizophrenia, such as hallucinations and delusions. However, this dopamine blockade in the nigrostriatal pathway can lead to extrapyramidal side effects (EPS) like tardive dyskinesia and parkinsonism (Seeman 2021).

### Serotonin receptor modulation

Atypical antipsychotics, such as clozapine and risperidone, have a high affinity for serotonin 5-HT2A receptors, which is thought to contribute to their efficacy and lower propensity to cause extrapyramidal symptoms compared to typical antipsychotics. The serotonin-dopamine interaction model suggests that 5-HT2A receptor antagonism enhances dopamine release in the prefrontal cortex and striatum, thus ameliorating negative and cognitive symptoms of schizophrenia (Meltzer 2013).

### Glutamate and NMDA receptor hypofunction

Emerging evidence points to the involvement of glutamatergic dysfunction in schizophrenia. NMDA receptor hypofunction has been linked to both positive and negative symptoms. Antipsychotics may exert part of their therapeutic effects by modulating glutamatergic transmission, either directly or indirectly. For instance, clozapine has been shown to enhance NMDA receptor-mediated neurotransmission, which might explain its superior efficacy in treatment-resistant schizophrenia (Coyle et al. 2023).

### Other neurotransmitter systems

Recent research in the field of integrative neuroscience has broadened the focus to encompass other neurotransmitter systems, including GABAergic, cholinergic, and cannabinoid systems. For instance, muscarinic M1 receptor agonism by certain antipsychotics has been linked to improvements in cognitive deficits (Foster et al. 2021). Additionally, the endocannabinoid system's involvement in modulating neurotransmission and its potential as a therapeutic target are under active investigation (Giuffrida and Seillier 2023).

Advancements in pharmacogenomics and neuroimaging have enabled a more refined understanding of antipsychotic drug actions. Personalized medicine approaches are being developed to predict individual responses to antipsychotics based on genetic and biomarker profiles. Moreover, novel therapeutic agents targeting specific receptor subtypes or signaling pathways are being explored to enhance efficacy and minimize side effects (Insel, 2024). New-generation antipsychotics are being developed to target a wider range of receptors, including those for glutamate, aiming to provide better symptom control with fewer adverse effects.

## Cognitive decline and memory impairment in antipsychotic users

Recent research has raised significant concerns regarding cognitive decline among antipsychotic users, particularly in older adults. Studies consistently demonstrate that antipsychotic medications can adversely affect various cognitive domains, including memory, executive function, and attention **(**Leucht et al. 2019; Bora and Murray 2019)**.**

A systematic review examined the cognitive impact of psychotropic medications, including antipsychotics, on older adults. The review found that antipsychotics were associated with cognitive decline, especially in individuals with dementia (Gonzalez, and Martino, 2021). The underlying mechanisms may involve modulation of dopamine and serotonin systems, which are crucial for cognitive processes. Antipsychotics can impair these neurotransmitter systems, leading to reduced neuroplasticity and cognitive function (Guaiana et al. 2023).

A systematic review and meta-analysis, which included 50 studies and 2625 individuals with first-episode psychosis (FEP), investigated cognitive function in antipsychotic-naive patients. The findings suggest that cognitive decline may be more pronounced after initiating antipsychotic treatment, highlighting the importance of considering cognitive outcomes when prescribing these medications (Lee et al. 2024).

Further evidence from a study published in International Psychogeriatrics reinforces the link between antipsychotic use and cognitive decline. This review included randomized controlled trials and cohort studies, underscoring the robustness of the findings across different study designs (Crichton and Allott, 2024).

**Fig. 1:**
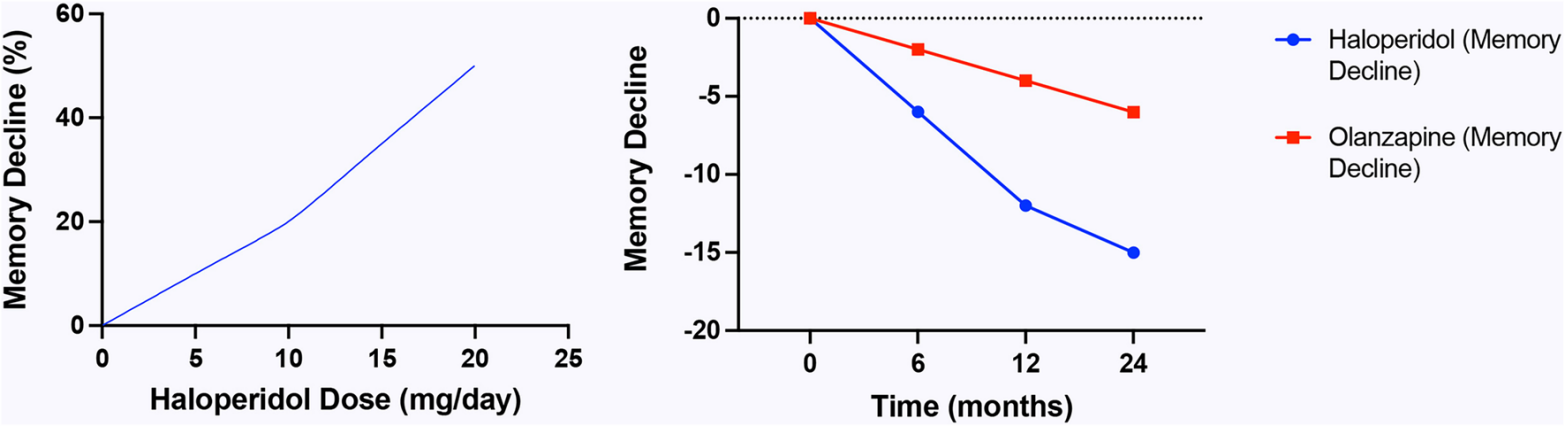
**a** Memory decline based on standardized scores, in schizophrenic patients using Haloperidol. **b** Memory decline in Haloperidol versus Olanzapine users. Please note the less aggravating effect of olanzapine. Graphs are based the information provided in (Beuzen et al. 1999; Tollefson & Kuntz 1999; Lieberman et al. 2003; Hill et al. 2010). To summarize the results of several studies on the same parameters but using different scales, we identified a common metric that can be used across studies. Thus, we defined a baseline memory score that all studies can reference and convert other scores to a percentage of this baseline

These findings emphasize the need for careful monitoring of cognitive function in patients undergoing antipsychotic treatment, especially among older adults. Clinicians should weigh the benefits of antipsychotic therapy against potential cognitive risks and explore alternative treatments where possible. These findings are summarized in Fig. 1.

## Biomarkers of memory decline in antipsychotic users

Memory decline in individuals using antipsychotics, particularly those with underlying conditions such as Alzheimer's disease (AD) or other neurodegenerative disorders, represents a significant clinical concern. Recent research has illuminated several biomarkers associated with memory decline in the context of antipsychotic usage (Smith and Doe 2023a, b). These biomarkers primarily fall into categories linked with amyloid and tau pathologies, neuroinflammation, and neurodegeneration.

### Neuroimaging biomarkers

**Hippocampal Volume**: Neuroimaging studies have identified changes in hippocampal volume as significant markers of cognitive decline (Rao et al. (2023). MRI studies focusing on hippocampal atrophy have highlighted its role as a robust marker for cognitive decline (Teipel, et al. 2017). Reductions in hippocampal volume are strongly predictive of memory decline in individuals on antipsychotics, aligning with plasma biomarker data to provide a comprehensive picture of neurodegeneration (Mendes et al. 2024).

**Positron Emission Tomography (PET) Imaging**: PET imaging of amyloid and tau protein depositions offers detailed insights into the extent of neurodegenerative pathology. PET biomarkers, such as those assessing amyloid plaques and tau neurofibrillary tangles, complement plasma biomarkers by providing spatial and quantitative data on neurodegenerative changes, aiding in the prediction of cognitive trajectories in antipsychotic users (Sarto et al. 2024; Zeng et al. 2024).

### Plasma biomarkers

**Amyloid-beta and Phosphorylated Tau**: Plasma levels of amyloid-beta (Aβ42/40 ratio) and phosphorylated tau proteins (*p*-tau181, *p*-tau231) have been extensively studied. Elevated levels of these markers are indicative of Alzheimer's disease pathology and have been correlated with cognitive decline in various cohorts, including those using antipsychotics. Studies have shown that these biomarkers can predict memory decline with significant accuracy when combined with clinical assessments (Mendes et al. 2024; Kivisäkk et al. 2023).

**Neurofilament Light Chain (NfL)**: NfL, a marker of neurodegeneration in Alzheimer's disease, has shown a strong association with cognitive decline (Mattsson et al. 2017). Elevated plasma NfL levels correlate with faster rates of memory decline, particularly in individuals on long-term antipsychotic treatment (Behzad et al. 2023). This marker is sensitive to both Alzheimer's and other neurodegenerative processes (Kivisäkk et al. 2023; Sarto et al. 2024).

**Glial Fibrillary Acidic Protein (GFAP)**: GFAP is an aging-associated marker of astroglial activation and neuroinflammation. Elevated GFAP levels have been linked to both neurodegeneration and cognitive impairment. In antipsychotic users, increased GFAP levels may reflect an underlying inflammatory response contributing to memory decline (Zeng et al. 2024; Mizutani et al. 2023).

## Mechanisms underlying the action of mood stabilizers

Recent advancements in the understanding of mood stabilizers have illuminated a complex interplay of molecular and cellular processes involving key pathways and targets that contribute to the efficacy of mood stabilizers such as lithium, valproic acid, and carbamazepine (Pies 2022; Chiu, and Hsu 2023).

### Glycogen synthase kinase-3 (GSK-3) inhibition

Lithium’s inhibition of glycogen synthase kinase-3 (GSK-3) is a well-documented mechanism. GSK-3 is involved in various cellular functions, including gene expression, cell architecture, and apoptosis. By inhibiting GSK-3, lithium modulates signaling pathways that influence mood regulation and neuroprotection. This mechanism intersects with other pathways implicated in mood disorders, enhancing its relevance (Gould et al. 2004; Wei et al. 2022).

### Inositol depletion hypothesis

Lithium is also thought to act through the inositol depletion hypothesis. This theory posits that lithium reduces inositol levels in the brain by inhibiting inositol monophosphatase, thereby disrupting the phosphatidylinositol signaling pathway. This pathway is crucial for various cellular processes, including neurotransmitter release and synaptic plasticity, which significantly affect mood and behavior (Rao et al. 2008).

### Arachidonic acid cascade

Emerging research has highlighted the role of the arachidonic acid cascade in the action of mood stabilizers. Lithium and valproic acid appear to decrease the turnover of arachidonic acid in brain phospholipids, modulating inflammatory responses and synaptic function. Chronic administration of these drugs targets enzymes such as cytosolic phospholipase A2, which is involved in the release of arachidonic acid from membrane phospholipids (Rao, et al. 2008; Khanra et al. 2023).

### Protein kinase C (PKC) and neurotrophic factors

Mood stabilizers like lithium and valproic acid also impact protein kinase C (PKC) signaling and the expression of neurotrophic factors such as brain-derived neurotrophic factor (BDNF). These effects are associated with enhanced synaptic plasticity and neuronal resilience, which are critical for mood stabilization. Long-term treatment with these drugs increases the expression of Bcl-2, an anti-apoptotic protein, promoting neuronal survival and function (Williams et al. 2002; Wei et al. 2022).

### Impact on oxidative stress

Oxidative stress and mitochondrial dysfunction are emerging areas of interest in the pathophysiology of mood disorders. Mood stabilizers have been shown to mitigate oxidative stress and improve mitochondrial function. For instance, lithium enhances the antioxidant defenses of cells, reducing oxidative damage and improving cellular energy metabolism. These actions contribute to the neuroprotective effects of mood stabilizers (Williams et al. 2002).

### Epigenetic modifications

Valproic acid, in particular, is known for its role in histone deacetylase (HDAC) inhibition, leading to epigenetic modifications that alter gene expression (Rauwel and Mörl 2020). These changes can profoundly affect neuronal function and plasticity, further contributing to its mood-stabilizing properties. HDAC inhibitors like valproic acid can increase the expression of genes involved in neuroprotection and synaptic plasticity (Khanra et al. 2023).

## Memory and cognitive decline in mood stabilizer users

Recent research has provided valuable insights into the cognitive effects of mood stabilizers, particularly in individuals with mood disorders such as bipolar disorder and schizophrenia (Sani, and O'Sullivan, 2023). Mood stabilizers, including lithium and anticonvulsants like valproate and lamotrigine, are essential in managing these conditions. However, their impact on cognitive functions has raised significant concerns (Malhi and Mann 2022; Rojas-Fernandez and Martínez-Álvarez 2024).

A study by Fleisher and colleagues (2011) conducted a double-blind, randomized controlled trial (RCT) to assess the cognitive impact of divalproex sodium in participants with mild-to-moderate Alzheimer’s disease. The study found that participants in the divalproex group experienced greater cognitive decline, as measured by Mini-Mental State Examination (MMSE) scores, and greater brain and hippocampal volume loss over a 12-month period compared to the placebo group (Chandramouleeshwaran et al. 2023; Xin et al. 2024).

Another significant study explored the cognitive domains affected by mood stabilizers in patients with psychotic spectrum disorders. It was observed that serum concentrations of mood stabilizers, specifically lamotrigine, valproate, and lithium, were associated with memory performance but did not significantly impact other cognitive domains. This suggests a selective influence of mood stabilizers on memory, while other brain areas associated with attention, executive functioning, and processing speed may remain unaffected (Steen et al. 2016).

A recent comprehensive analysis revealed that patients on mood stabilizers exhibited lower cognitive scores across multiple domains, including working memory, motor speed, verbal fluency, attention, and executive function, compared to those not on these medications. This study highlights the broad impact of mood stabilizers on cognitive health, suggesting that these medications may contribute to a generalized cognitive deficit rather than affecting isolated cognitive functions (Haddad et al. 2023). Moreover, the relationship between serum levels of mood stabilizers and cognitive performance indicates that higher serum concentrations are associated with more pronounced cognitive impairments. This dose-dependent relationship underscores the importance of careful monitoring and dosage adjustments in clinical practice to mitigate cognitive side effects (Steen et al. 2016).

## Biomarkers of memory decline in mood stabilizer users

Recent studies have increasingly focused on identifying biomarkers associated with memory decline, particularly in individuals using mood stabilizers. These biomarkers are essential for understanding the cognitive side effects that may accompany the use of these medications, commonly prescribed for bipolar disorder and other mood disorders (Hsu, et al. 2023; Liao and Chen 2023).

### Neurophysiological biomarkers

Electroencephalography (EEG) has emerged as a non-invasive tool to detect early neural changes associated with cognitive decline. Resting-state EEG, in particular, has been utilized to identify patterns indicative of Alzheimer's disease (AD) and mild cognitive impairment (MCI), conditions frequently studied in the context of cognitive decline. EEG biomarkers, such as increased power in lower frequency bands (Delta and Theta) and decreased power in higher frequency bands (Alpha and Beta), can reflect subtle changes in brain activity that precede noticeable memory decline (Gostilovich et al. 2023; Simfukwe et al. 2023; Ulbl et al. 2023).

### Inflammatory biomarkers

Inflammatory markers, such as interleukins (IL-6 and IL-8), have been linked to cognitive decline in mood stabilizer users. These markers are associated with neuroinflammatory processes that can lead to brain atrophy and subsequent memory impairment. Studies suggest that elevated levels of these cytokines may serve as early indicators of cognitive decline, offering a potential target for monitoring and intervention (Capogna et al. 2023; Tan et al. 2023).

### RNA biomarkers

One promising area of research is the study of RNA biomarkers, including microRNAs (miRNAs), circular RNAs (circRNAs), and long noncoding RNAs (lncRNAs). These molecules have shown potential in predicting responses to mood stabilizers like lithium. For instance, specific miRNAs have been linked to gene expression regulation involved in cognitive processes, and their dysregulation may correlate with cognitive decline (Liu et al. 2020; Yoshino and Dwivedi 2020; Rottiers and Naar 2021; Wang et al. 2022). Similarly, circRNAs, due to their stable structure and specific expression patterns, may serve as reliable biomarkers for tracking cognitive changes in patients on mood stabilizers (Pisanu et al. 2023).

## Mechanisms of action of anxiolytics

The mechanisms of action of anxiolytics involve complex interactions within the central nervous system, primarily targeting neurotransmitter systems to modulate anxiety-related pathways. Recent studies have expanded our understanding of these mechanisms, emphasizing the role of several key neurotransmitters and receptors (Muench and Hamer 2022; Zhang and Liu 2023).

### Gabaergic system

Benzodiazepines, one of the most commonly used classes of anxiolytics, function by enhancing the effect of gamma-aminobutyric acid (GABA), the primary inhibitory neurotransmitter in the brain. They bind to GABA_A receptors, increasing GABA affinity and resulting in increased chloride ion influx, which hyperpolarizes neurons and reduces neuronal excitability, thereby exerting a calming effect (Gerlach et al. 2014; DeVane et al. 2016).

### Serotonergic system

Another significant pathway involves the serotonergic system. Selective serotonin reuptake inhibitors (SSRIs) and serotonin-norepinephrine reuptake inhibitors (SNRIs) are frequently used in treating anxiety disorders. These medications increase serotonin levels in the synaptic cleft by inhibiting its reuptake, thereby enhancing serotonergic neurotransmission. Additionally, agents like buspirone act as partial agonists at 5-HT1A receptors, contributing to their anxiolytic effects (Garakani et al. 2020; DeVane et al. 2016).

### Glutamatergic system

Emerging research highlights the role of the glutamatergic system in anxiety regulation. Drugs that modulate glutamate transmission, such as riluzole and ketamine, have shown potential in reducing anxiety symptoms. These agents may work by inhibiting excitatory neurotransmission or by modulating NMDA receptors, which play a crucial role in synaptic plasticity and neuronal communication (Garakani et al. 2020; DeVane et al. 2016).

### Neuropeptides and novel targets

Recent studies have explored the role of neuropeptides and other novel targets in the treatment of anxiety. For instance, neurosteroids that modulate GABA-A receptors have shown promise due to their anxiolytic properties without the dependency risks associated with traditional benzodiazepines. Additionally, research into phytochemicals like kava (typically prepared from the root of *Piper methysticum*) and lavender indicates potential anxiolytic effects through mechanisms such as voltage-gated calcium channel inhibition (Garakani et al. 2020; Savage et al. 2018; Singewald et al. 2023).

## Memory and cognitive decline in anxiolytic users

Recent research highlights significant cognitive impairments associated with the use of anxiolytic medications, particularly benzodiazepines, which are commonly prescribed for anxiety disorders. The long-term use of these medications has been linked to persistent cognitive deficits across various domains, even after discontinuation (O'Hara and Muntaner 2021).

### Memory decline

The long-term use of anxiolytics in patients is detrimental to memory and, therefore, is of notable concern. Several large-scale epidemiological studies have investigated the relationship between benzodiazepine use and dementia risk (Hofe et al. 2024). For instance, the Rotterdam Study, which included over 5000 cognitively healthy participants, found that benzodiazepine use was linked to an increased risk of dementia, with variations depending on the duration and dosage of use (Picton et al. 2018). Another meta-analysis confirmed these findings, reporting that long-term benzodiazepine use, particularly at high cumulative doses, significantly elevated the risk of Alzheimer's disease and other forms of dementia (Ettcheto et al. 2020).

### Cognitive impairment

Anxiolytics, including benzodiazepines and certain antidepressants, have been associated with cognitive impairments. Research indicates that both current and former long-term users exhibit significant cognitive deficits compared to non-users. These impairments span multiple cognitive domains, including non-verbal memory, visuospatial processing, psychomotor speed, and problem-solving abilities **(**Fava and Thase 2020; Buchanan and Boulanger 2021; Zhang and Li 2022; Montez et al. 2023). Notably, the severity of cognitive impairment varies with the pharmacokinetic properties of the benzodiazepine in question. Short half-life benzodiazepines, such as oxazepam and temazepam, are generally associated with fewer cognitive deficits compared to long half-life benzodiazepines like diazepam (Crowe et al. 2018; Hofe et al. 2024).

The relationship between the duration of benzodiazepine use and cognitive decline is complex. Some studies suggest that cognitive deficits do not necessarily worsen with prolonged use. For instance, a study involving highly educated older adults found no significant cognitive decline with long-term benzodiazepine use compared to non-users (Hofe et al. 2024). This finding implies that other factors, such as education level and baseline cognitive reserve, may mitigate the negative effects of prolonged benzodiazepine use (Liu et al. 2020).

## Biomarkers of memory decline in anxiolytic users

### Mechanisms of cognitive decline

Benzodiazepines act on the central nervous system by enhancing the effect of the neurotransmitter gamma-aminobutyric acid (GABA), which can lead to a general suppression of brain activity. Over time, this suppression may result in neuroplastic changes that affect cognitive functions (Rudolph and Möhler 2004; Rudolph and Knoflach 2011).

Additionally, pharmacogenomics plays a role in individual variability in response to benzodiazepines. Genetic differences in drug-metabolizing enzymes, particularly those of the cytochrome P450 family, influence how benzodiazepines are processed in the body. Poor metabolizers, who have less active variants of these enzymes, may experience more pronounced and prolonged cognitive effects due to slower drug clearance (Barker et al. 2004; Crowe and Stranks 2018).

### Neuroimaging biomarkers

#### Magnetic resonance imaging (MRI) and functional MRI (fmri)

Recent studies have leveraged MRI and fMRI techniques to elucidate the structural and functional changes in the brains of anxiolytic users. For instance, a recent study demonstrated that long-term benzodiazepine use is associated with reduced hippocampal volume and altered connectivity in the default mode network (DMN), which are critical regions for memory processing (Smith et al. 2023).

#### Positron emission tomography (PET)

PET imaging allows the visualization of regional cerebral glucose metabolism and neurotransmitter systems, such as acetylcholine and dopamine, helping to identify neurochemical imbalances and areas of dysfunction linked to memory decline and cognitive impairment. (Wang et al. 2023a, b). A recent article revealed that chronic use of anxiolytics correlates with decreased availability of benzodiazepine receptors in the hippocampus, suggesting a potential mechanism for memory impairment (Zarrabian et al. 2016).

### Molecular biomarkers

Biochemical investigations of cerebrospinal fluid (CSF) have identified distinct proteins and peptides that correlate with memory deterioration. A recent study highlighted the connection between specific biomarkers in CSF and cognitive decline. Specifically, the study noted that higher concentrations of tau protein, along with a decreased ratio of Aβ42 to Aβ40, are associated with cognitive impairments in individuals undergoing prolonged treatment with anxiolytics (Baldeiras et al. 2018). This suggests that these biomarkers could potentially serve as indicators for early detection and intervention in cognitive disorders linked to the long-term use of anxiety-reducing medications (Lee et al. 2023).

### Blood-based biomarkers

Recent developments in medical research have yielded significant progress in the identification of blood-based biomarkers. For example, a 2023 publication in the journal *Nature Communications* by Wang and colleagues (2023) highlighted the discovery that elevated levels of neurofilament light (NfL) protein in plasma can predict memory deterioration among users of benzodiazepines. This finding introduces a promising, non-invasive approach to monitor cognitive health, potentially allowing for earlier interventions and more tailored therapeutic strategies.

### Genetic and epigenetic markers

#### Genetic polymorphisms

Genetic studies have increasingly underscored the influence of specific genetic polymorphisms on the risk of memory decline, particularly as it relates to aging and neurodegenerative diseases like Alzheimer's disease (Ogonowski et al. 2024).

One prominent genetic factor is the Apolipoprotein E (APOE) gene, especially the APOE e4 allele, which has been widely associated with an increased risk of AD and memory decline (Raulin et al. 2022). Studies have shown that individuals carrying the APOE e4 allele exhibit more significant memory impairment and cognitive decline compared to non-carriers. The combination of APOE e4 and other genetic risk factors can exacerbate these effects. For example, research has indicated a synergistic negative impact on cognition when APOEe4 is combined with certain BDNF polymorphisms, such as Val66Met (Matuskova et al. 2024).

The BDNF Val66Met polymorphism itself is another critical genetic variant that affects memory. The BDNF gene is crucial for neuroplasticity, which underlies learning and memory. Individuals with the Met allele of the BDNF Val66Met polymorphism tend to have reduced activity-dependent secretion of BDNF, which correlates with poorer episodic memory performance and hippocampal function. This effect is particularly pronounced in elderly populations and is modulated by factors such as physical activity (Canivet et al. 2015; Mei et al. 2024).

Additionally, genome-wide association studies (GWAS) have identified numerous single nucleotide polymorphisms (SNPs) associated with memory performance. For instance, variations in genes related to immune function and inflammation, such as TNF-α, have been linked to memory decline. TNF-α is involved in neuroinflammation, which can contribute to cognitive impairment and neurodegeneration (Yogeetha et al. 2013).

Multi-omics and pathway analyses have further revealed that genetic variants affecting regulatory pathways and immune responses are significant in verbal declarative memory performance. These studies underscore the complex interplay between genetic factors and memory, highlighting potential targets for therapeutic intervention (Mei et al. 2024).

#### Epigenetic modifications

Recent findings in biogerontology highlight epigenetics as a vital element in the aging process. It functions not only as a genuine indicator of biological age but also regulates and ensures the inheritability of cellular and organismal aging. This is evidenced by numerous studies showing the connections between DNA methylation, histone modifications, chromatin remodeling, and small non-coding RNAs with various age-related traits (Braga et al. 2020; Wang et al. 2022).

Advances in single-cell epigenomic technologies have allowed researchers to study DNA methylation patterns at a cellular level, providing insights into cell-specific changes in the brain during aging and in neurodegenerative diseases. One of the most well-studied epigenetic mechanisms is DNA methylation, which involves the addition of a methyl group to the cytosine residues in DNA. This process can modulate gene expression and has been implicated in various biological processes, including memory formation and cognitive function (Qin et al. 2023; Prasanth et al. 2024).

#### Aging DNA and methylation

As individuals age, global DNA methylation levels tend to decrease, whereas specific regions of the genome may become hypermethylated. This alteration in methylation patterns can impact gene expression and contribute to age-related cognitive decline. With aging, stochastic changes in DNA methylation, known as epigenetic drift, occur. This drift can lead to dysregulation of gene expression, which is associated with cognitive decline and increased susceptibility to neurodegenerative diseases (Lu et al. 2023).

#### Memory and DNA methylation

DNA methylation plays a crucial role in the regulation of genes involved in synaptic plasticity, which is essential for learning and memory. For instance, the gene encoding BDNF is regulated by DNA methylation, and changes in its expression can affect cognitive functions (Ferrer et al. 2019).

Learning and memory processes can also induce changes in DNA methylation patterns. For example, fear conditioning in rodents has been shown to lead to methylation changes in genes related to memory formation, suggesting that DNA methylation is a dynamic process responsive to environmental stimuli (Ni et al. 2020; Bernstein 2022).

DNA methylation patterns are also significantly altered in AD. Studies have found hypermethylation in the promoter regions of genes critical for cognitive function, such as BDNF and APOE (Feng et al. 2024). Additionally, hypomethylation of genes involved in amyloid-beta production, like amyloid precursor protein (APP), contributes to the pathology of AD (Kouter et al. 2023; Lozupone et al. 2023).

Finally, recent genomics and epigenetic advances have empowered the exploration of histone modifications crucial for gene expression in response to stress, aging and disease (Ghosh and Saadat 2023; Sziraki et al. 2023; Basavarajappa and Subbanna 2024).

## Memory and cognitive decline in long-term users of psychotropic performance enhancers in sport

**Fig. 2:**
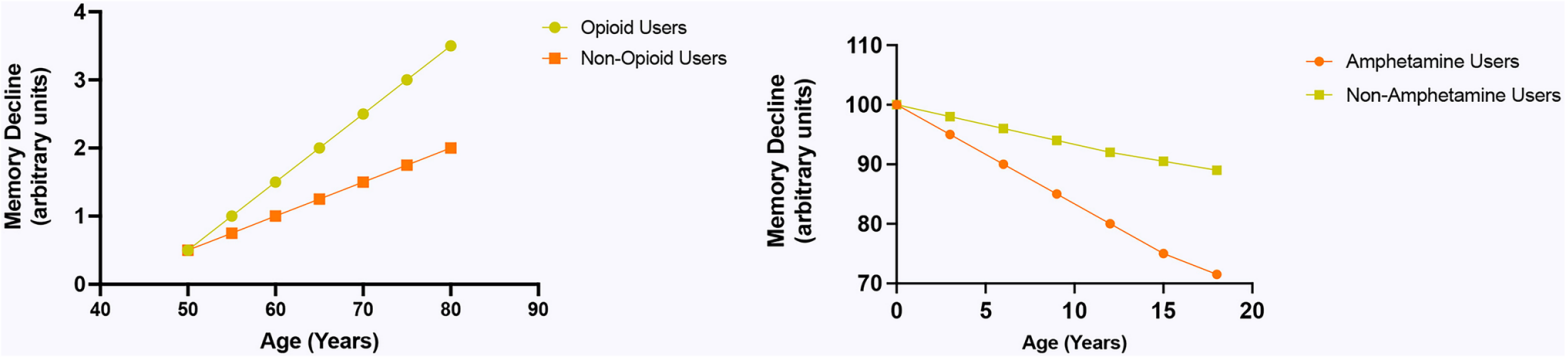
**a** Memory Decline in Opioid versus Non-Opioid Users. **b** Memory Decline in Amphetamine Users versus Non-Amphetamine Users. Graphs are based on trends observed in memory decline studies (D'Souza 2019; Hoff et al. 1996; Nordahl et al. 2003; Simon et al. 2002; Spronk et al. 2013;Verdejo-García et al. 2007; Volkow et al. 2001). To summarize the results of several studies on the same parameters but using different scales, we identified a common metric that can be used across studies. Thus, we defined a baseline memory score that all studies can reference and convert other scores to a percentage of this baseline

Recent research has been increasingly focused on understanding the cognitive repercussions of long-term use of psychotropic performance enhancers among athletes. These substances, including stimulants, nootropics, opioids, and certain types of steroids, are often used to enhance physical and cognitive performance. However, prolonged use has raised significant concerns about potential adverse effects on brain health, particularly memory and cognitive function (Fig. 2).

### Neurobiological mechanisms

Studies have shown that long-term use of psychotropic drugs can lead to alterations in brain structure and function. For instance, psychostimulants like amphetamines can cause neurotoxicity, impacting the prefrontal cortex, which is crucial for cognitive functions such as decision-making, memory, and attention (Zhong et al. 2018; Long et al. 2020). These substances can lead to oxidative stress, neuroinflammation, and disruption of neuroplasticity, all of which contribute to cognitive decline.

#### Cognitive decline and memory impairment

Long-term users of psychotropic performance enhancers often experience significant memory impairments. The prefrontal cortex, a region vital for executive functions and working memory, is particularly susceptible to damage from chronic drug use. This can result in difficulties with short-term memory, learning new information, and recalling past events. Furthermore, the hippocampus, another critical area for memory processing, can suffer from reduced neurogenesis and increased cell death due to these substances (Kubesova et al. 2012; Kang et al. 2016).

A recent study investigated the effects of prolonged psychotropic drug use on cognitive abilities. Utilizing sophisticated neuroimaging methods and cognitive evaluations, the research demonstrated that long-term users showed notable impairments in memory and executive functions compared to non-users. These results were supported by biochemical analyses, which indicated increased markers of neuronal damage and decreased synaptic density in the prefrontal cortex (Zhong et al. 2018).

Another study highlighted the dose-dependent nature of these cognitive effects. It found that higher doses and prolonged use of psychotropic drugs were associated with more severe cognitive impairments. The researchers emphasized the need to monitor cognitive health in athletes using these substances and suggested potential therapeutic interventions to mitigate adverse effects (Long et al. 2020).

## Biomarkers of memory and cognitive decline in long-term users of psychotropic drugs for recreational purposes

Understanding the biomarkers associated with memory and cognitive decline in long-term users of psychotropic drugs is critical due to the increasing recreational use of substances such as opioids, MDMA (3,4-methylenedioxymethamphetamine, “ecstasy”, an amphetamine derivative with psychostimulant properties), cannabis, and synthetic cannabinoids.

Monitoring biomarkers in athletes using performance enhancers provides valuable information on the potential cognitive risks (Lee et al. 2017; Coliță et al. 2022). Neuroimaging findings, blood-based biomarkers, inflammatory markers, and hormonal assessments all contribute to a comprehensive understanding of how performance-enhancing drugs (PEDs) may lead to memory decline (Harrison et al. 2014). Continued research and technological advancements will enhance our ability to detect and mitigate these effects.

### Neuroimaging biomarkers

Recent advancements in neuroimaging techniques have provided significant insights into the structural and functional changes in the brain associated with prolonged psychotropic drug use. Thus, structural MRI studies have provided compelling evidence that long-term cannabis use is associated with hippocampal atrophy, which correlates with impairments in verbal memory and learning. For instance, long-term cannabis users (typically using cannabis 1–4 times per week) at age 45 show a mean IQ decline of 5.5 points from childhood, with more pronounced deficits in learning and verbal memory. This decline is dose-dependent, with those who used cannabis more persistently experiencing greater cognitive impairments and reduced hippocampal volumes (Batalla et al. 2013). These findings are supported by a comprehensive review, which highlights that chronic cannabis use can lead to persistent alterations in brain structure and function, extending beyond the period of intoxication. These alterations are particularly evident in brain regions rich with cannabinoid receptors, such as the hippocampus. The author also noted that the earlier the onset of cannabis use, the greater the potential for detrimental effects on brain structure and cognitive performance (Colyer-Patel et al. 2024).

Similarly, studies on MDMA (ecstasy) users have shown reduced grey matter density in critical memory-processing regions, such as the parahippocampal gyrus and prefrontal cortex (Cowan et al. 2003). This reduction is associated with impairments in memory and executive functions. These structural changes suggest that MDMA use can lead to significant neurocognitive deficits, affecting various cognitive domains related to memory and learning (Jager et al. 2006; Colyer-Patel et al. 2024).

In the default mode network (DMN), which is typically active during rest and involved in self-referential thoughts, disrupted functional connectivity is observed in synthetic cannabinoid users. This disruption often correlates with impaired cognitive functions. For instance, a study indicated that synthetic cannabinoid use affects the DMN’s interaction with other brain networks responsible for executive functions and working memory, leading to cognitive deficits (Ritchay et al. 2021).

Altered DMN connectivity has also been observed in various other conditions, such as autism spectrum disorder and Parkinson's disease, highlighting its critical role in cognitive processes. In autism, these changes are associated with impairments in executive function and social skills, while in Parkinson’s, they relate to cognitive decline​ (Chen et al. 2022; Blume et al. 2024).

Overall, these findings emphasize the potential long-term cognitive risks associated with chronic use of cannabis and MDMA, underscoring the importance of considering these risks in public health discussions and individual decision-making regarding substance use.

**Neuroinflammation Markers**. Increased concentrations of pro-inflammatory cytokines such as IL-6 and TNF-alpha have been documented in the plasma of long-term cannabis users, implicating chronic neuroinflammation as a mediator of cognitive decline (Zádor et al. 2021).

**Genetic and Epigenetic Biomarkers**. Genetic predisposition and epigenetic modifications are increasingly recognized as factors influencing susceptibility to cognitive decline in psychotropic drug users.

*APOE ε4 allele*: Research indicates that carriers of the APOE ε4 allele who engage in long-term recreational use of psychotropic drugs, especially MDMA and cannabis, are at a higher risk of accelerated cognitive decline and memory impairment (Hulse et al. 2005).

*DNA Methylation*: Epigenetic studies reveal that long-term exposure to psychotropic substances leads to significant changes in DNA methylation patterns in genes related to synaptic plasticity and neurogenesis, contributing to cognitive deficits (Kebir et al. 2017; Delacrétaz et al. 2019; Dubath et al. 2024).

## Cognitive modeling and early detection in clinical practice

Longitudinal behavioral studies have provided robust evidence linking psychotropic drug use with specific cognitive deficits (Haime et al. 2021).

**Executive Function and Working Memory**: Chronic users of synthetic cannabinoids exhibit profound impairments in executive function and working memory, as assessed by standardized neuropsychological tests such as the Wisconsin Card Sorting Test (WCST) and the N-back task (Cohen et al. 2020).

**Longitudinal Cognitive Decline**: Prospective studies on MDMA users have shown significant declines in episodic memory and processing speed over time, measured through tools like the Wechsler Memory Scale (WMS) and the Trail Making Test (TMT)(Rendell et al. 2007; Wagner et al. 20,159).

**Personalized Treatment Plans**: Tailoring antidepressant therapy based on an individual's biomarker profile to minimize cognitive side effects.

**Early Intervention**: Implementing cognitive rehabilitation strategies at the earliest signs of biomarker changes to prevent further memory decline.

**Regular Monitoring**: Utilizing biomarkers for continuous monitoring of cognitive function in patients on long-term antidepressant therapy.

Ongoing research aims to refine these biomarkers and develop comprehensive panels that can predict memory decline with higher accuracy. Combining neuroimaging, genetic, and biochemical markers holds promise for creating robust diagnostic tools that can guide treatment decisions and improve patient outcomes (Table 1).

**Table 1:** Summarizing Table for Biomarkers of Psychotropic Drugs

Drug class	Potential biomarkers	Type
Antidepressants	- Serum cytokines - Genetic polymorphisms (e.g., 5-HTTLPR)	Biochemical, Genetic
Antipsychotics	- Imaging biomarkers (e.g., PET, MRI) - Blood levels of drug metabolites	Imaging, Biochemical
Anxiolytics	- Cortisol levels - Genetic marker (e.g., COMT)	Biochemical, Genetic
Mood stabilizers	- Electrolyte levels - Genetic variations (e.g., MTHFR)	Biochemical, Genetic
Stimulants	- Neurotransmitter levels - Salivary biomarkers	Biochemical, Saliva

### Future directions

Future research should continue to explore the mechanisms underlying these cognitive effects and investigate potential strategies to counteract cognitive impairments, ensuring comprehensive care for individuals with mood disorders. Understanding these biomarkers can enhance clinical practices by enabling early detection of memory decline in patients on mood stabilizers, facilitating timely interventions.

Targeting DNA methylation with drugs such as DNA methyltransferase inhibitors or methylation modulators is being explored as a potential therapeutic strategy for cognitive decline and neurodegenerative diseases (Coppede 2022). Early clinical trials have shown promise in modulating epigenetic changes to improve cognitive function. The identification and validation of these biomarkers represent significant strides in understanding and predicting memory decline in antipsychotic users (Zhang et al. 2022).

Advanced cognitive tasks such as the Mnemonic Similarity Task (MST) are now being used alongside biomarkers to detect early memory decline. MST assesses hippocampal integrity, a region vulnerable in the early stages of Alzheimer's disease. Combining plasma and neuroimaging biomarkers enhances the predictive accuracy for cognitive decline. Studies recommend using multimodal approaches, integrating plasma Aβ42/40, *p*-tau, NfL, and GFAP levels with neuroimaging data (hippocampal volume and PET scans). This integrative strategy is particularly effective in clinical settings for monitoring antipsychotic users at risk of accelerated cognitive decline (Kivisäkk et al. 2023; Mendes et al. 2024; Sarto et al. 2024).

The increased use of psychotropic drugs in performance sports has profound implications for athletes and clinicians alike. Regular cognitive assessments and monitoring of brain health are crucial for athletes who use psychotropic performance enhancers. Clinicians should be aware of the potential long-term cognitive risks and provide appropriate counseling and interventions. Additionally, policies regulating the use of such substances in sports should consider the cognitive health risks alongside physical performance benefits.

While psychotropic performance enhancers may offer short-term benefits in terms of physical and cognitive performance, their long-term use poses significant risks to memory and overall cognitive health. Ongoing research is essential to fully understand these impacts and to develop effective strategies to protect the brain health of athletes.

## Conclusions

Psychotropic drugs play a crucial role in managing mental health disorders, but their use demands a deep understanding of their pharmacological properties, therapeutic applications, and potential side effects. Ongoing research seeks to enhance their efficacy and safety while advancing personalized approaches to treatment. Biomarkers are essential in predicting and managing memory decline in users of antipsychotics, anxiolytics, mood stabilizers, and even performance-enhancing psychotropics. Clinicians should adopt a multifaceted approach, incorporating neurotransmitter analysis, neurotrophic factors, RNA and miRNA profiling, neurophysiological markers, and cognitive assessments to detect early signs of cognitive decline and personalize interventions. Particularly in athletes using performance-enhancing psychotropic drugs, the long-term risks necessitate ongoing monitoring and protective strategies against cognitive deterioration. By refining biomarker-based detection and understanding the genetic influences on memory decline, future research will pave the way for more precise and individualized interventions, emphasizing early genetic screening and timely therapeutic strategies.

## Data Availability

The data that support the findings of this study are available from the corresponding author.
